# Co-administration of *Lactobacillus gasseri* KBL697 and tumor necrosis factor-alpha inhibitor infliximab improves colitis in mice

**DOI:** 10.1038/s41598-022-13753-6

**Published:** 2022-06-10

**Authors:** Dae Hee Han, Woon-ki Kim, Cheonghoon Lee, SungJun Park, Kiuk Lee, Sung Jae Jang, GwangPyo Ko

**Affiliations:** 1 Department of Environmental Health Sciences, Graduate School of Public Health, Seoul National University, Seoul, Republic of Korea; 2 Institute of Health and Environment, Seoul National University, Seoul, Republic of Korea; 3 N-Bio, Seoul National University, Seoul, Republic of Korea; 4 KoBioLabs, Inc, Seoul, Republic of Korea

**Keywords:** Immunology, Microbiology, Health care

## Abstract

Inflammatory bowel disease (IBD) refers to disorders involving chronic inflammation of the gastrointestinal tract. Well-established treatments for IBD have not yet to be suggested. To address this gap, we investigated the effects of co-administration of *Lactobacillus gasseri* (*L. gasseri*) KBL697 and infliximab (IFX), the first approved tumor necrosis factor (TNF)-alpha inhibitor, on the dextran sodium sulfate-induced colitis mouse model. 2 × 10^9^ colony-forming units/g of *L. gasseri* KBL697 were administered to seven-week-old female C57BL/6J mice daily by oral gavage. On day three, IFX (5 mg/kg) suspended in 1 × PBS (200 µL) was intravenously injected in the IFX-treated group and all mice were sacrificed on day nine. Co-administration of *L. gasseri* KBL697 and IFX improved colitis symptoms in mice, including body weight, disease activity index, colon length, and histology score. Additionally, pro-inflammatory cytokines, such as interferon-gamma, interleukin (IL)-2, IL-6, IL-17A, and TNF were significantly decreased, while IL-10, an anti-inflammatory cytokine, was increased. Expression levels of tight junction genes and CD4 + CD25 + Foxp3 + T regulatory cells in the mesenteric lymph nodes were synergistically upregulated with the combined treatment. Furthermore, co-administered mice displayed altered cecum microbial diversity and composition with increases in the genus *Prevotella*. Related changes in the predicted amino and nucleic acid metabolic pathways were also evident, along with increased acetate and butyrate level. Therefore, the synergistic effect of *L. gasseri* KBL697 and IFX co-administration is a possible method of prevention and treatment for IBD.

## Introduction

Inflammatory bowel disease (IBD) is an umbrella term describing chronic inflammatory disorders in the gastrointestinal tract^1^. Well-established treatments for IBD have not yet to be suggested^2^. Recent evidence has indicated that disruption of the homeostasis between the mucosal immune system in gut and gut microbiota correlates with increased susceptibility to IBD^3^. Gut microbiota can affect integrity of the gut mucosal barrier and immunomodulation of host^4,5^. Additionally, short-chain fatty acids (SCFAs) synthesized by gut microbiota have key roles in adjusting host metabolism and regulating immune response^6^. Previous studies have suggested that changes in bacterial composition of the gut can lead inflammation^7^.

Tumor necrosis factor-alpha (TNF-α), an important pro-inflammatory cytokine, is a major immune-cell activator and involved in immunopathology of IBD^8^. Therefore, inhibition of TNF-α has been suggested as a novel treatment for IBD. For example, infliximab (IFX), a chimeric mouse–human monoclonal antibody, can effectively neutralize human TNF-α and has been used in alleviating IBD symptoms^9^. Another suggested treatment for IBD is probiotics. Probiotics have been shown to be useful in the prevention and treatment for various acute and chronic human diseases^10^. Probiotics can interact with the intestinal epithelium and modulate immune system of host^11–13^. Previous studies have been especially suggested that *Lactobacillus* spp*.* can alleviate IBD symptoms via modulation of gut microbiota compositions, metabolic pathways and immune systems of host^12,14^.

Tight junction proteins have important roles for gut permeability. During the colon inflammation, the expression of tight junction proteins can be altered and epithelial mucosa is disrupted, which can cause invasion of pathogens^15,16^. *Lactobacillus* spp. can affect the improvement of mucus layer through the recovery of intestinal microbiota^17,18^. Moreover, our previous studies have been suggested that administration of various isolates of *Lactobacillus* spp., including* Lactobacillus fermentum* and *Lactobacillus paracasei*, have strong immunomodulation effects, can improve tight junction protein expression and maintain the diversity of gut microbiota in the colitis-induced mice^4,11^.

Therefore, in this study, we investigated the effect of co-administration of *Lactobacillus gasseri* (*L. gasseri*) KBL697, isolated from the vaginal samples of healthy donors, and IFX for treatment in dextran sodium sulfate (DSS)-induced colitis mouse model. First, we induced the acute colitis in mice and co-treated *L. gasseri* KBL697 and IFX. We assessed amelioration of IBD symptoms and abnormal immune responses due to *L. gasseri* KBL697 and IFX. Moreover, we confirmed microbial compositions and their predicted metabolic pathways in colon of mice with colitis due to co-treatment of *L. gasseri* KBL697 and IFX. We also evaluated the levels of acetate and butyrate, important SCFAs produced by genus *Lactobacillus*, to elucidate of possible mechanisms of co-administration of *L. gasseri* KBL697 and IFX for IBD treatment.

## Methods

### Bacteria strain preparation

Lyophilized *L. gasseri* KBL697 [2 × 10^10^ colony-forming unit (CFU)/g], isolated from vaginal samples of healthy Korean adults, were provided by KoBioLabs, Inc (Seoul, Republic of Korea). We confirmed superior resistances of *L. gasseri* KBL697 to high concentration of bile salts and low pH (data not shown). The bacteria were diluted appropriately using 1 × phosphate-buffered saline (PBS) before administration.

### In vivo colitis mouse model

We developed the in vivo colitis mouse model as previously described^11,12^. Briefly, seven week-old female C57BL/6J mice (Central Lab Animals Inc., Seoul, Republic of Korea) were purchased for the colitis model. All experimental procedures were approved by the Institutional Animal Care and Use Committee (IACUC: SNU-190528-1) of Seoul National University, Republic of Korea and in compliance with ARRIVE guidelines. All procedures were carried out in accordance with relevant guidelines and regulations. Five groups of mice (n = 8) [(1) Water + PBS, (2) DSS + PBS, (3) DSS + IFX, (4) DSS + KBL697, and (5) DSS + KBL697 + IFX (DSS + Combine)] were kept in air-conditioned cages and fed the water containing 2% DSS (molecular mass 36,000–50,000 Da; MP Biomedical, LLC., Santa Ana, CA, USA) for seven days to induce acute colitis. The group treated with water + PBS were used as the negative control. Simultaneously, 200 μL of 1 × PBS with 2 × 10^9^ CFU of *L. gasseri* KBL697 were administered daily by oral gavage. On day three, IFX (5 mg/kg) suspended in 1 × PBS (200 µL) was intravenously injected for the IFX-treated group. Disease activity index (DAI) of mice were measured daily using the following criteria: (1) weight loss (%), (2) stool consistency and (3) blood in feces, as previously described (Table S1)^11,12^. On day nine, the mice were sacrificed and organs, including colon, cecum, and mesenteric lymph nodes (MLNs), and stool samples were collected for further experiments.

### Histological analysis

Distal colon samples were fixed in 10% formaldehyde and stained with hematoxylin and eosin, as described previously^19^. Tissues were analyzed using a panoramic viewer (3DHISTECH, Ltd., Budapest, Hungary). Histological scores were measured using the following criteria: (1) loss of epithelium, (2) crypt damage, (3) depletion of goblet cells and (4) inflammatory cell infiltration (Table S2)^20^. Three individual researches separately evaluated all samples and averaged scores were suggested.

### Myeloperoxidase (MPO) measurement

Colon samples were weighed and homogenized in 1 × radioimmunoprecipitation assay (RIPA) buffer (Thermo Fisher Scientific, Waltham, MA, USA) with the Halt protease inhibitor cocktail (Thermo Fisher Scientific) using a Mixer Mill MM 400 homogenizer (Retsch, GmbH, Haan, Germany), as described previously^11,12^. Homogenates were centrifuged at 4 °C for 10 min at 15,000×*g* and supernatants were collected. MPO concentration in the supernatant was measured using an ELISA kit (Hycult Biotech. Inc., Plymouth Meeting, PA, USA) according to the manufacturer’s instructions.

### Cytokine measurement

Levels of various cytokines in the supernatant, including interferon (IFN)-γ, interleukin (IL)-2, IL-6, IL-10, IL-17A, and TNF, were measured using a BD Cytometric Bead Array Mouse T helper cell (Th)1/Th2/Th17 Cytokine Kit (BD Biosciences, San Jose, CA, USA) according to the manufacturer’s instructions.

### mRNA measurement related to tight junction

Total RNA was extracted from distal colon sample using an easy-spin total RNA Extraction Kit (Intron, Seoul, Republic of Korea) according to the manufacturer’s instructions. RNA samples were quantified using a Nanodrop ND 1000 (Thermo Fisher Scientific) and reverse-transcribed using a High-Capacity RNA-to-cDNA kit (Thermo Fisher Scientific). cDNA samples were amplified using a Power SYBR Green PCR Master Mix (Thermo Fisher Scientific) and specific primers designed for the tight junction-related genes, including zonula occludens (ZO)-1, ZO-2, and Claudin4 (Table 1)^7,21^. Quantitative reverse transcription polymerase chain reaction (qRT-PCR) were performed using a Step-One-Plus Real-Time PCR System (Applied Biosystems, Forster, CA USA) with the following conditions: initially denatured at 95 °C for 10 min; 40 cycles of 95 °C for 15 s and 60 °C for 60 s. mRNA levels were normalized using hypoxanthine–guanine phosphoribosyltransferase (Hprt) gene^22^.

**Table 1:** Primers used in this study.

Gene	Sequence (5' → 3')	References
ZO-1	Fw: 5′-ACC CGA AAC TGA TGC TGT GGA TAG-3′	^21^
	Rv: 5′-AAA TGG CCG GGC AGA ACT TGT GTA-3′	
ZO-2	Fw: 5′-CTA GAC CCC CAG AGC CCC AGA AA-3′	^21^
	Rv: 5′-TCG CAG GAG TCC ACG CAT ACA AG-3′	
Claudin4	Fw: 5′-ACT TTT TGT GGT CAC CGA CT-3′	^21^
	Rv: 5′-GCG AGC ATC GAG TCG TAC AT-3′	
Hprt	Fw: 5′-TTA TGG ACA GGA CTG AAA GAC-3′	^7^
	Rv: 5′-GCT TTA ATG TAA TCC AGC AGG T-3′	

*Fw* Represents sequences of a forward primer.

*Rv* Represents sequences of a reverse primer.

### T regulatory cell analysis

To analyze T regulatory cells (Tregs), flow cytometry analysis was performed as described previously^11,12^. MLNs were smashed carefully and filtered using a cell strainer (100 μm pore diameter, SPL Life Science Co., Ltd., Pocheon-Si, Gyeonggi-do, Republic of Korea). Live cells were stained with a Fixable Viability Stain 510 (FVS510; BD Biosciences, San Jose, CA, USA) and the cell surface was stained using anti-CD3 fluorescein isothiocyanate (145-2C11; BD Biosciences), anti-CD4 Percep-Cyanine5.5 (RM4-5; BD Biosciences), and anti-CD25 phycoerythrin (PC61; BD Bioscience), according to the manufacturer’s instructions. Cells were permeabilized with a fixation/permeabilization buffer (eBioscience, San Diego, CA, USA), and subjected to intracellular staining using an Alexa Fluor 647 anti-Foxp3 antibody (MF23; BD Biosciences). Finally, CD4 + CD25 + Foxp3 + Tregs were measured using a BD FACSVerse Flow Cytometer (BD Biosciences). Immunoglobulin G isotypes (BD Biosciences) were used as a control.

### Cecal microbiota analysis

Analysis for cecal microbiota was performed as described previously^11,12^. Briefly, total bacterial DNA in cecum was extracted using a QIAamp Fast DNA Stool Mini Kit (Qiagen), according to the manufacturer’s instructions, and the V4-5 region of 16S rRNA gene was amplified using the universal primers (515F and 926R)^23^. PCR amplicons were sequenced appropriately using an Illumina Miseq platform (Illumina Inc., San Diego, CA, USA). The Quantitative Insights into Microbial Ecology (QIIME)2 version 2022.2 (QIIME 2 Development Team; https://qiime2.org/) with Greengenes version 13_8 database (http://greengene.secondgenome.com) were used for data analysis^24,25^. Low-quality or duplicated, and chimeric sequences were removed using Divisive Amplicon Denoising Algorithm 2 (DADA2). Sequences were classified into operational taxonomic units at the 99% similarity level. Alpha-diversity was indicated using Faith’s phylogenetic diversity (PD) whole tree index while beta-diversity was calculated using unweight UniFrac distance and demonstrated three-dimentional plots. The Linear discriminant analysis (LDA) Effect Size analysis (LEfSe) were performed using Galaxy ver. 2.1.1 (Hutlab; http://huttenhower.org/galaxy) as described previously^11,12^. Phylogenetic investigation of communities by reconstruction of unobserved states (PICRUSt)2 analysis (version 2.4.1) was also performed using QIIME 1.8.0 (QIIME Development Team; http://qiime.org) with Greengenes version 13_8 database and the Kyoto Encyclopedia of Genes and Genomes pathway database (GenomeNet; https://www.genome.jp/kegg/pathway.html)^11,12^.

### Short-chain fatty acid measurement

SCFAs in cecum samples were analyzed as described previously^11,12^. Briefly, cecum was well-dispersed in 1 mL distilled water by vigorous vortexing and the supernatant was collected by centrifugation for 5 min at 13,000 ×*g*. 2-methylpentanoic acid (1%) was added as the internal standard. Then, ethyl ether was applied as the extraction solvent. After centrifugation for 5 min at 13,000 ×*g*, the organic layer was collected and analyzed using an Agilent 7890A gas chromatograph (Agilent Technologies, Santa Clara, CA, USA) as described previously^11,12^. SCFA standards were used as references for retention time analysis and quantification of sample peak areas^26^.

### Statistical analysis

Data are expressed as means ± standard deviation of the experimental groups in three independent experiments. GraphPad Prism ver. 5.04 (GraphPad Software, Inc., La Jolla, CA, USA) was applied for statistical analyses, including one-way analysis of variance (ANOVA) with Dunnett’s post hoc test and visualizations. P-values (*P*) < 0.05 were considered statistically significant.

## Results

### Effects of *L. gasseri* KBL697 and IFX co-administration on colitis symptoms of mice

**Figure 1:**
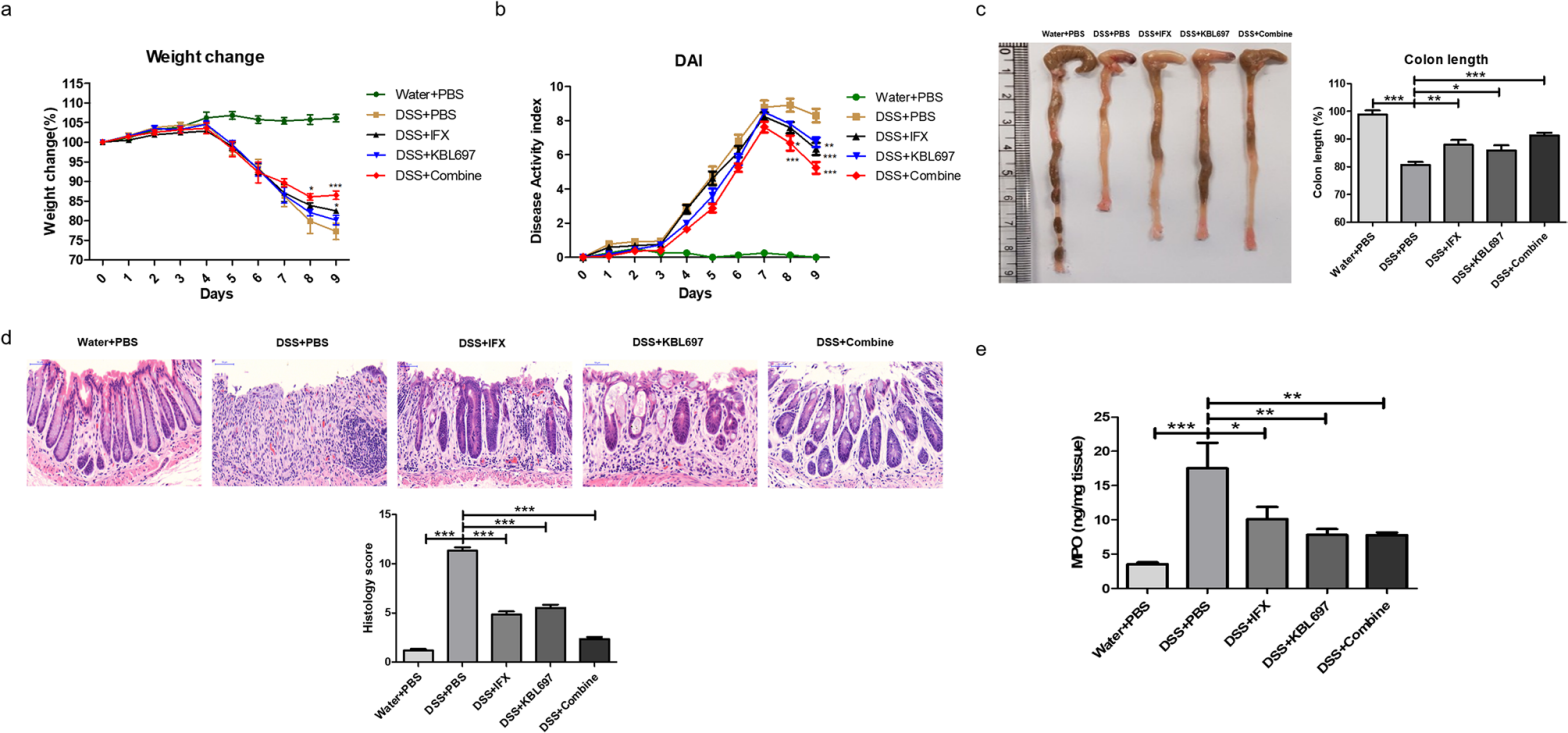
Improvements of colitis symptoms in mice via co-administration of *L. gasseri* KBL697 and Infliximab (IFX). (**a**,**b**) Body weights and DAI scores measured during experimental period. (**c**,**d**) Colon lengths and histology scores. (**e**) MPO levels. Statistical analysis was performed using one-way ANOVA with Dunnett’s post hoc test (*P < 0.05; **P < 0.01; ***P < 0.001).

Figure 1 shows improvements of colitis symptoms via co-administration of *L. gasseri* KBL697 and IFX. Compared to the DSS + PBS group, body weights and DAI scores of DSS + Combine-treated mice were clearly restored after treatment (*P* < 0.001) (Fig. 1a,b). Moreover, DSS + Combine-treated mice showed significantly longer colon lengths (*P* < 0.001) and higher histology scores (*P* < 0.001) compared to DSS + PBS-treated mice (Fig. 1c,d). MPO in colon tissues collected from DSS + Combine and DSS + KBL697-treated mice were also significantly decreased compared to the DSS + PBS group (*P* < 0.01) (Fig. 1e).

### Differences in the cytokine levels of mice with DSS-induced colitis using co-administration

**Figure 2:**
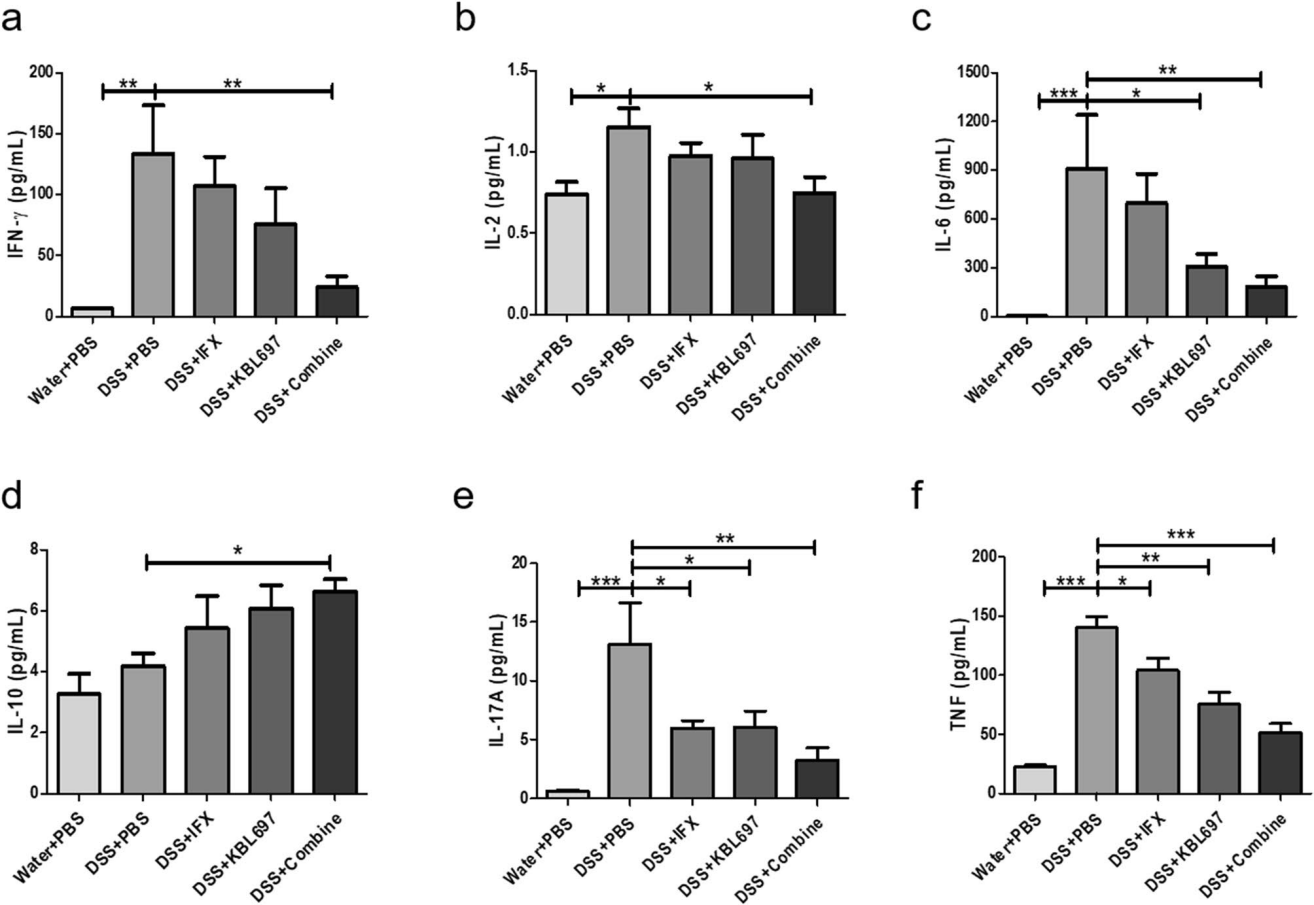
Effect of the different treatments on colon cytokine levels. (**a**) IFN-γ. (**b**) IL-2. (**c**) IL-6. (**d**) IL-10. (**e**) IL-17A. (**f**) TNF. Statistical analysis was performed using one-way ANOVA with Dunnett’s post hoc test (*P < 0.05; **P < 0.01; ***P < 0.001).

Figure 2 shows the effects of co-administration on cytokine levels of the mice with DSS-induced colitis. Overall, only the co-administered group showed significant decrease of IFN-γ, IL-2 and IL-6 levels compared to the DSS + PBS group (*P* < 0.05) (Fig. 2a–c). Moreover, high levels anti-inflammatory cytokine IL-10 in DSS + Combine-treated mice was discovered compared to the DSS + PBS group (*P* < 0.05) (Fig. 2d). Additionally, the co-administered group had the lowest IL-17A (*P* < 0.01) and TNF (*P* < 0.001) levels among all experimental groups (Fig. 2e,f).

### Effects of co-administration in the mRNA expressions related to tight junction

**Figure 3:**
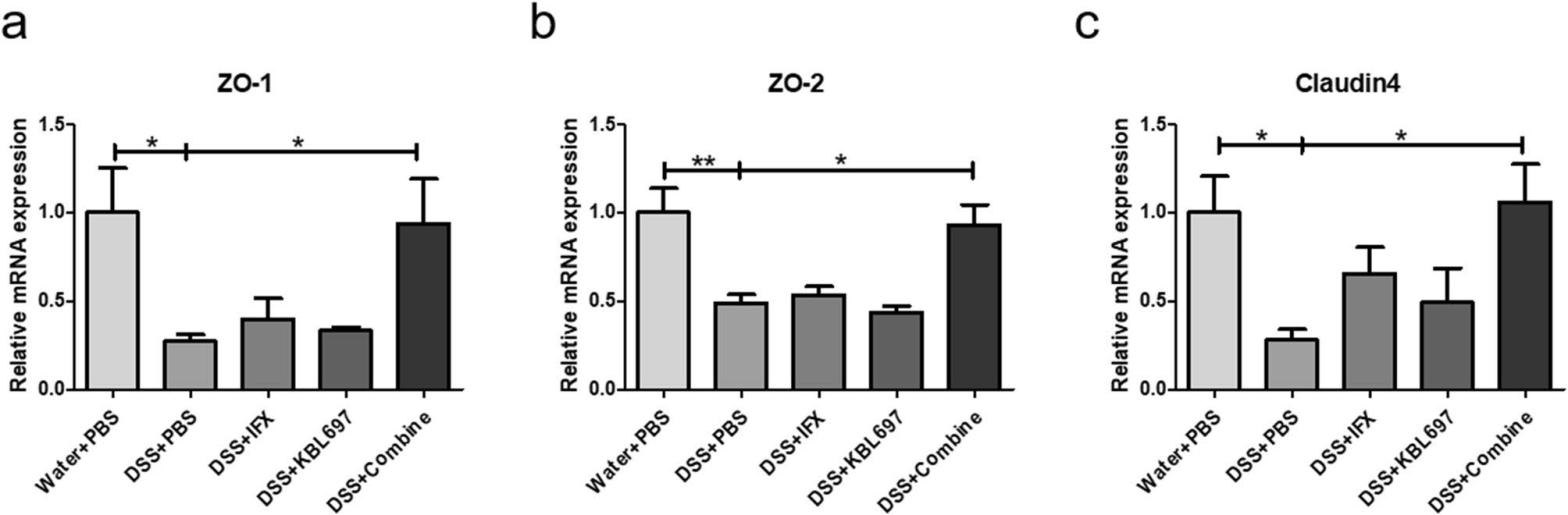
Effects of the different treatments on the mRNA expressions related to tight junction. (**a**) ZO-1. (**b**) ZO-2. (**c**) Claudin4. Statistical analysis was performed using one-way ANOVA with Dunnett’s post hoc test (*P < 0.05; **P < 0.01; ***P < 0.001).

DSS + Combine-treated mice showed significant restoration of mRNA expressions for ZO-1, ZO-2 and Claudin4 compared to the DSS + PBS group (*P* < 0.05) (Fig. 3). DSS + IFX and DSS + KBL697-treated mice showed higher mRNA level of Claudin4 than the DSS + PBS group, however, there were no statistical significance discovered (Fig. 3c).

### Effects of co-administration on CD4 + CD25 + Foxp3 + Tregs in MLNs of mice with DSS-induced colitis

**Figure 4:**
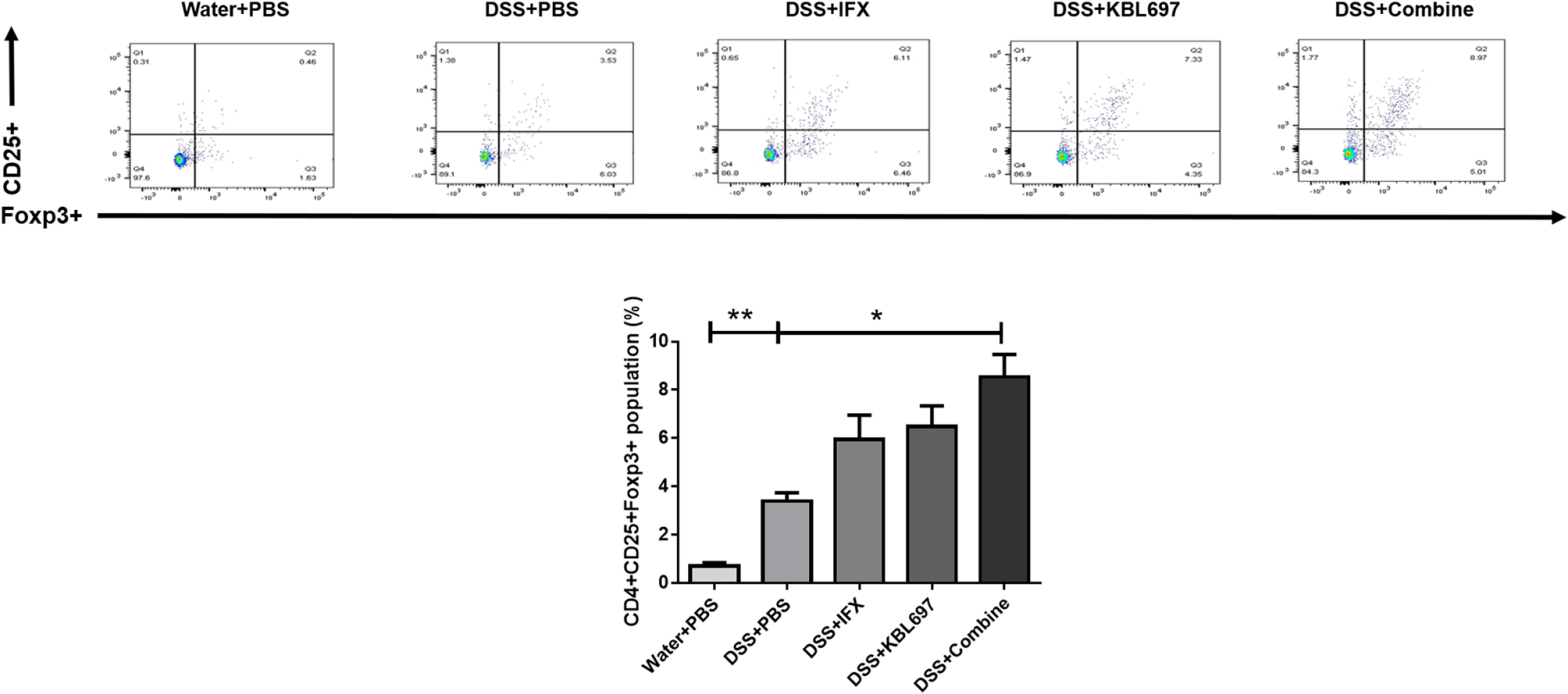
Effect of the different treatments on CD4 + CD25 + Foxp3 + Tregs. Statistical analysis was performed using one-way ANOVA with Dunnett’s post hoc test (*P < 0.05; **P < 0.01).

Figure 4 shows that CD4 + CD25 + Foxp3 + Tregs were significantly increased in DSS + Combine-treated mice compared to the DSS + PBS group (*P* < 0.05) (Fig. 4). DSS + IFX and DSS + KBL697-treated mice also showed higher CD4 + CD25 + Foxp3 + Tregs than the DSS + PBS group, however, there were no statistical significance discovered (Fig. 4).

### Effects of *L. gasseri* KBL697 and IFX co-administration on cecal microbiota

**Figure 5:**
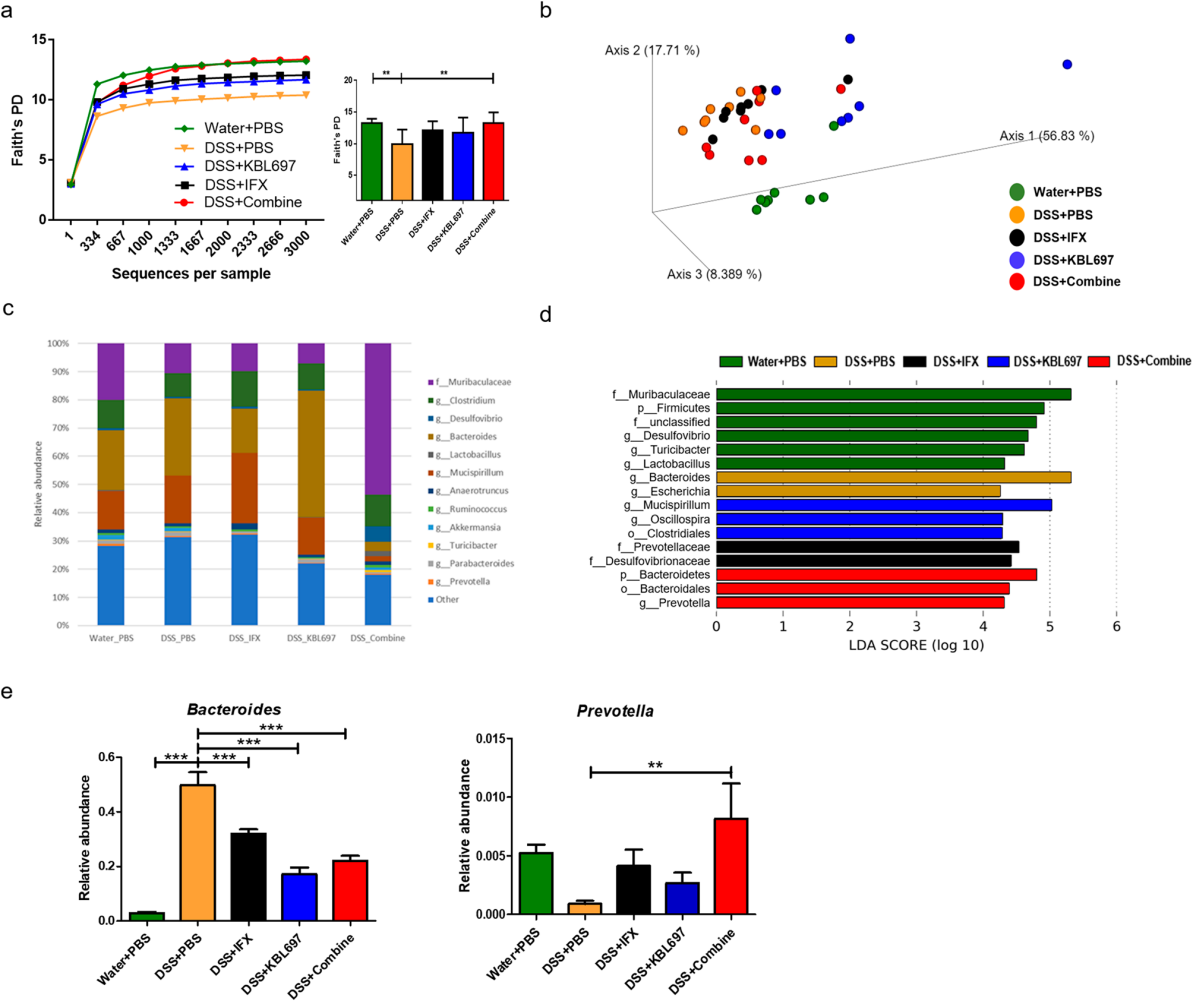
Effect of the different treatments on cecal microbiota composition. (**a**) Rarefaction plots using Faith’s phylogenetic diversity (PD) whole tree indices. (**b**) Principal coordinates analysis with unweighted UniFrac distances. (**c**) Taxonomic structures of cecal microbiota. (**d**) LEfSe results in experimental groups (threshold > 2.5). (**e**) Relative abundances of significantly different microbial taxa in experimental groups at the genus level. When appropriate, statistical analysis was performed using one-way ANOVA with Dunnett’s post hoc test (*P < 0.05; **P < 0.01; ***P < 0.001).

DSS + Combine-treated mice have significantly increased bacterial diversities compared to the DSS + PBS group (*P* < 0.01). The bacterial communities of DSS + Combine-treated mice only showed similarity (did not shown significantly differences) with the DSS + IFX group (Fig. 5a,b). Unlike the Water + PBS group, which has family *Muribaculaceae* as their major cecal microbiota, genus *Bacteroides* was the dominant in DSS + PBS-treated mice (Fig. 5c,d). However, the microbial profiles for DSS + Combine-treated mice showed the clear increase in family *Muribaculaceae* and decrease in genus *Bacteroides* (Fig. 5c,d). Additionally, the higher relative abundances of genus *Prevotella* (*P* < 0.01) were discovered in DSS + Combine-treated mice compared to the DSS + PBS group (Fig. 5e).

### Differences in predicted profiles of functional alteration related to cecal microbiota and SCFAs via co-administration

**Figure 6:**
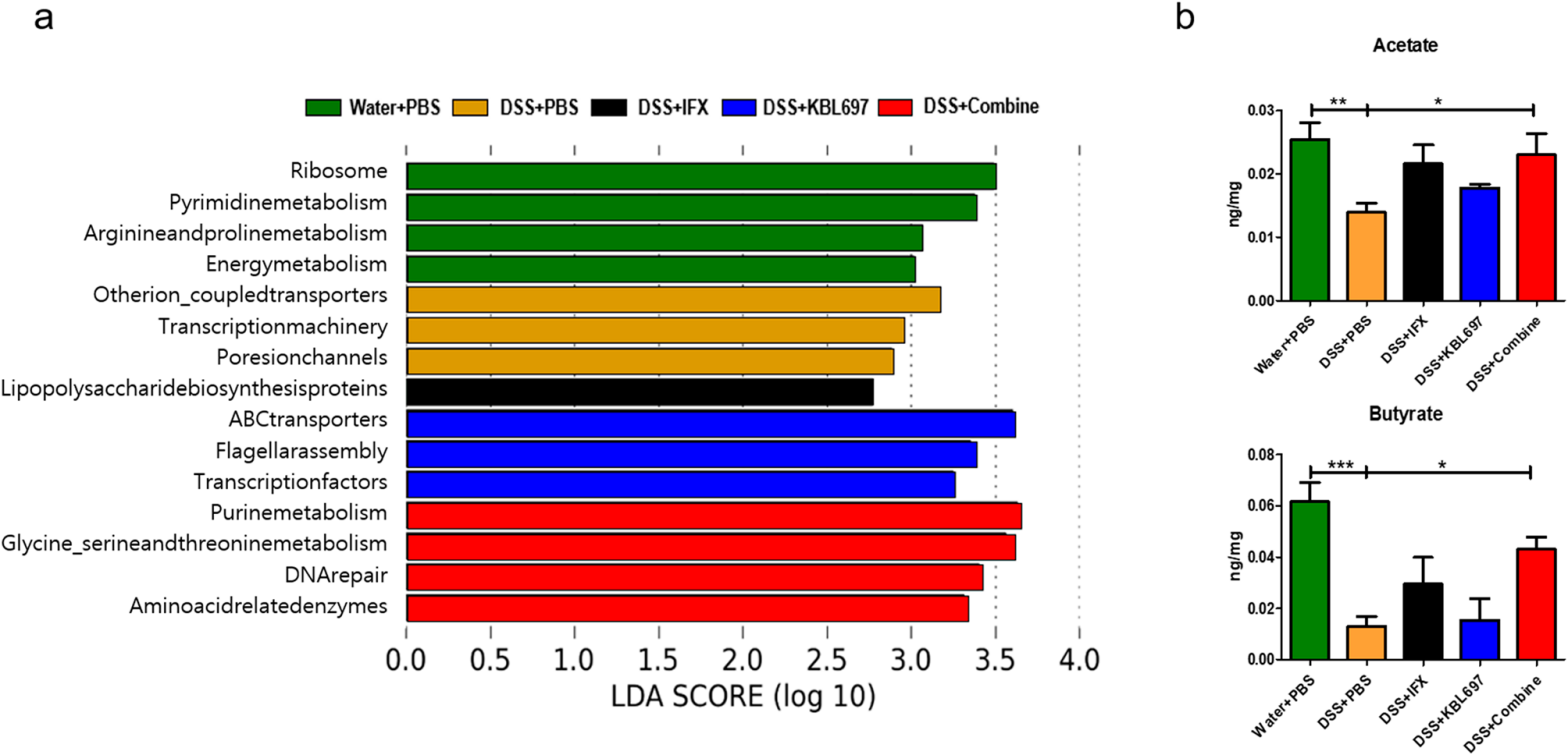
Differences in profiles of functional alteration related to cecal microbiota and SCFAs via the different treatments. (**a**) PICRUSt results in experimental groups (threshold > 2.5). (**b**) Acetate and butyrate levels. When appropriate, statistical analysis was performed using one-way ANOVA with Dunnett’s post hoc test (*P < 0.05; **P < 0.01; ***P < 0.001).

Figure 6a presents the predicted functional alterations due to cecal microbial composition in the various treatment groups. The microbiota in Water + PBS-treated mice displayed strong correlations with metabolic pathways related to amino acid, energy, and nucleic acid. Similar to the Water + PBS group, the microbiota in the DSS + Combine-treated mice were highly correlated with metabolic pathways related to amino acid and nucleic acid and they had correlation with DNA repair pathway. Moreover, significant increases of acetate and butyrate (*P* < 0.05) were discovered in DSS + Combine-treated mice (Fig. 6b).

## Discussion

In this study, we preliminarily confirmed that co-administration of *L. gasseri* KBL697 and IFX, a commercialized TNF-α inhibitor, can significantly alleviate colitis symptoms in the DSS-induced colitis mouse model. Moreover, levels of MPO, which is the important inflammatory mediator of innate immunity, were clearly restored after co-administration^11,12^. Especially, the effects for co-administration in colitis symptoms were clearly higher than single treatment of *L. gasseri* KBL697 or IFX, indicating that co-administration has synergistic effects for prevention and treatment of colitis. Activation of innate immune cells lead to the development of antigen-specific T cells, such as Th1 and Th17, during the acute stage of DSS-induced colitis and can exacerbate colitis severely^27–29^. We observed that the levels of pro- or inflammatory cytokines, such as IFN-γ, IL-2, IL-6, IL-17A, and TNF, were appropriately controlled with co-administration. Moreover, co-administration resulted in a significant increase in the anti-inflammatory cytokine IL-10, which can aid in the treatment of colitis. However, in this study, we applied PBS as the negative control, hereafter, further studies should be applied using a suitable control of IFX, such as an equivalent intravenous injection of human immunoglobulin G1.

Tight junction proteins are important for establishing the gut epithelial barrier. ZO-1 and ZO-2 are intracellular tight junction proteins, which are the important linkers between cell cytoskeleton and transmembrane tight junction proteins^21^. Claudin4, an important transmembrane epithelial protein, also have a role in establishing integrity of the intestinal barrier^21^. Previous studies have been reported that probiotics or IFX treatment can improve intestinal permeability and promote colonic recovery^11,21^. Gene expression of tight junction proteins ZO-1, ZO-2, and Claudin4, were significantly up-regulated in DSS + Combine-treated mice. Therefore, co-administration of *L. gasseri* KBL697 and IFX can synergistically alleviate colitis by improving intestinal permeability. Further researches should be performed with cross-validation of various house-keeping genes to secure the accuracy of gene expression data. Moreover, to fully elucidate the KBL697 effects in attenuation of colitis, various experiments such as protein expression or localization related to tight junction need to be suggested.

Activated Tregs can suppress the progression of colitis effectively via production of inflammatory cytokines such as IL-10 and regulation of Th17s^29^. Co-administration of *L. gasseri* KBL697 and IFX increased CD4 + CD25 + Foxp3 + Tregs in MLNs of DSS-treated mice. Especially, our results suggested that the effects of co-administration are significantly higher than those of either single treatment of *L. gasseri* KBL697 or IFX, indicating that synergistic effects can be occurred.

A previous study suggested that the perturbation of microbial diversity and composition can accelerate inflammation in IBD patients^30^. In this study, we confirmed cecal microbiota composition, which is generally used to gut microbiome studies for mouse because mouse cecum is relatively larger than human and has digestion function for food components^31,32^. Feces is not suitable for our study because only watery-type feces can be collected from mice with DSS-induced colitis. We administered high concentration (2 × 10^9^ CFU/day) of *L. gasseri* KBL697 by oral gavage to mice and only mice in the Water + PBS group showed high abundance of *Lactobacillus* spp. in their cecal microbiome. Therefore, we can confirm indirectly that most administered *L. gasseri* KBL697 were excreted from digestive system of mouse with DSS-induced colitis. Our results showed that the microbial diversity was significantly different between the DSS + Combine-treated group and the DSS + PBS-treated group. The relative abundances of several bacterial species, including genus *Prevotella*, were also significantly increased in DSS + Combine-treated mice. Genus *Prevotella* can induce Tregs and regulate cytokines for suppression of diseases such as inflammation of central nervous system and multiple sclerosis^33^. Differences in microbial composition due to co-administration of *L. gasseri* KBL697 and IFX in mice with DSS-induced colitis can affect the predicted functional alterations of cecal microbiota, including metabolic pathways related to amino acid and nucleic acid, and production of important SCFAs such as acetate and butyrate. Acetate has various crucial roles for regulating epithelial cell differentiation, Treg stimulation, and amelioration of mucosal inflammation. Additionally, butyrate can exert beneficial effects on colitis treatment via induction of Tregs^34^. However, further longitudinal studies with various colitis models should be performed to fully elucidate the combination effects of *L. gasseri* KBL697 and IFX on gut microbiota and their functional alterations from IBD patients. Also, recent study was reported that mucus microbiota of mice has distinctive compositions compared to cecal samples or feces^35^, therefore, further studies using various gut samples with other reference microbiome databases such as SILVA rRNA database project (https://www.arb-silva.de/) or The Ribosomal Database Project (RDP; http://rdp.cme.msu.edu/) can be suggested important information for colonization or function of *L. gasseri* KBL697 in gut.

In conclusion, our study suggested that the co-administration of *L. gasseri* KBL697 and IFX exhibits synergistic effects for alleviation of colitis in mice via immunomodulation and restoration of gut microbiota. Further, additional studies for comparisons with widely used probiotic strains, such as *L. rhamnosus* GG^14^, need to be performed for development of the promising method of prevention and treatment of IBD.

## Supplementary Information

Supplementary Information.

## References

[1] Abraham C, Cho JH (2009). Inflammtory bowel disease. N. Engl. J. Med..

[2] Lügering A, Lebiedz P, Koch S, Kucharzik T (2006). Apoptosis as a therapeutic tool in IBD?. Ann. N. Y. Acad. Sci..

[3] Sartor RB (2008). Microbial influences in inflammatory bowel diseases. Gastroenterology.

[4] Kim WK (2019). Administration of *Lactobacillus paracasei* strains improves immunomodulation and changes the composition of gut microbiota leading to improvement of colitis in mice. J. Funct. Foods.

[5] Kim WK (2019). Administration of *Lactobacillus fermentum* KBL375 causes taxonomic and functional changes in gut microbiota leading to improvement of atopic dermatitis. Front. Mol. Biosci..

[6] Vonk RJ, Priebe M, Meijer K, Venema K, Roelofsen H (2011). The interaction of short-chain fatty acids (SCFA) with adipose tissue; relevance for systemic inflammation. Gastroenterology.

[7] Kim W (2020). *Lactobacillus paracasei* KBL382 administration attenuates atopic dermatitis by modulating immune response and gut microbiota. Gut Microbes.

[8] Macdonald TT, Monteleone G, Pender SLF (2000). Recent developments in the immunology of inflammatory bowel disease. Scand. J. Immunol..

[9] Scallon BJ, Moore MA, Trinh H, Knight DM, Ghrayeb J (1995). Chimeric anti-tnf-α monoclonal antibody ca2 binds recombinant transmembrane tnf-α and activates immune effector functions. Cytokine.

[10] Liu Y, Tran DQ, Rhoads JM (2019). Probiotics in disease prevention and treatment. J. Clin. Pharmacol..

[11] Jang YJ, Kim WK, Han DH, Lee K, Ko G (2019). *Lactobacillus fermentum* species ameliorate dextran sulfate sodium-induced colitis by regulating the immune response and altering gut microbiota. Gut Microbes.

[12] Kim W (2020). Alleviation of DSS-induced colitis via *Lactobacillus acidophilus* treatment in mice. Food Funct..

[13] Han DH, Kim WK, Park SJ, Jang YJ, Ko G (2020). *Lactobacillus paracasei* treatment modulates mRNA expression in macrophages. Biochem. Biophys. Rep..

[14] Ghouri YA (2014). Systematic review of randomized controlled trials of probiotics, prebiotics, and synbiotics in infammatory bowel disease. Clin. Exp. Gastroenterol..

[15] Poritz LS (2007). Loss of the tight junction protein ZO-1 in dextran sulfate sodium induced colitis. J. Surg. Res..

[16] Strober W, Fuss IJ, Blumberg RS (2002). The immunology of mucosal models of inflammation. Annu. Rev. Immunol..

[17] Round JL, Mazmanian SK (2009). The gut microbiota shapes intestinal immune responses during health and disease. Nat. Rev. Immunol..

[18] van Baarlen P, Wells JM, Kleerebezem M (2013). Regulation of intestinal homeostasis and immunity with probiotic lactobacilli. Trends Immunol..

[19] Ghaleb AM, McConnell BB, Kaestner KH, Yang VW (2011). Altered intestinal epithelial homeostasis in mice with intestine-specific deletion of the Krüppel-like factor 4 gene. Dev. Biol..

[20] Akgun E (2005). Effects of N-acetylcysteine treatment on oxidative stress in acetic acid-induced experimental colitis in rats. J. Int. Med. Res..

[21] Bischoff SC (2014). Intestinal permeability—a new target for disease prevention and therapy. BMC Gastroenterol..

[22] Rodríguez-Nogales A (2017). Differential intestinal anti-inflammatory effects of *Lactobacillus fermentum* and *Lactobacillus salivarius* in DSS mouse colitis: Impact on microRNAs expression and microbiota composition. Mol. Nutr. Food Res..

[23] Walters W (2015). Improved bacterial 16S rRNA gene (V4 and V4–5) and fungal internal transcribed spacer marker gene primers for microbial community surveys. MSystems.

[24] Caporaso JG (2012). Ultra-high-throughput microbial community analysis on the Illumina HiSeq and MiSeq platforms. ISME J..

[25] Caporaso JG (2010). QIIME allows analysis of high-throughput community sequencing data. Nat. Methods.

[26] David LA (2014). Diet rapidly and reproducibly alters the human gut microbiome. Nature.

[27] Morgan ME (2013). New perspective on dextran sodium sulfate colitis: Antigen-specific T cell development during intestinal inflammation. PLoS ONE.

[28] Brand S (2009). Crohn’s disease: Th1, Th17 or both? The change of a paradigm: New immunological and genetic insights implicate Th17 cells in the pathogenesis of Crohn’s disease. Gut.

[29] Boehm F (2012). Deletion of Foxp3+ regulatory T cells in genetically targeted mice supports development of intestinal inflammation. BMC Gastroenterol..

[30] Ni J, Wu GD, Albenberg L, Tomov VT (2017). Gut microbiota and IBD: Causation or correlation?. Nat. Rev. Gastroenterol. Hepatol..

[31] Nguyen TL, Vieira-Silva S, Liston A, Raes J (2015). How informative is the mouse for human gut microbiota research?. Dis. Model. Mech..

[32] Alessandro T (2017). Metaproteogenomics reveals taxonomic and functional changes between cecal and fecal microbiota in mouse. Front. Microbiol..

[33] Mangalam A (2017). Human gut-derived commensal bacteria suppress CNS inflammatory and demyelinating disease. Cell Rep..

[34] Machiels K (2014). A decrease of the butyrate-producing species roseburia hominis and *Faecalibacterium prausnitzii* defines dysbiosis in patients with ulcerative colitis. Gut.

[35] Kozik AJ, Nakatsu CH, Chun H, Jones-Hall YL (2019). Comparison of the fecal, cecal, and mucus microbiome in male and female mice after TNBS-induced colitis. PLoS ONE.

