# Supplementary material for: Co-administration of Lactobacillus gasseri KBL697 and tumor necrosis factor-alpha inhibitor infliximab improves colitis in mice
Source: Sci Rep. 2022 Jun 10;12:9640. doi: 10.1038/s41598-022-13753-6 (PMC9187735; doi:10.1038/s41598-022-13753-6)
Supplement: Supplementary file 1 — Supplementary Information. [file 41598_2022_13753_MOESM1_ESM.docx]

**Table S1** Calculated disease activity index (DAI) scores.

| **Score** | **Weight loss (%)** | **Stool consistency** | **Blood in feces** |
| --- | --- | --- | --- |
| 0 | None | Normal | Negative (no bleeding) |
| 1 | 1.0-5.0 | ­ | ­ |
| 2 | 5.0-10.0 | Loose stools | Positive (slight bleeding) |
| 3 | 10.0-15.0 | ­ | ­ |
| 4 | Over 15.0 | Watery diarrhea | Gross bleeding |

**Table S2** Histological scores of dextran sulfate sodium (DSS)-induced colitis.

| **Histological features** | **Score** | **Description** |
| --- | --- | --- |
| **Epithelium loss (%)** | 0 | None |
|  | 1 | 0.0-5.0 |
|  | 2 | 5.0-10.0 |
|  | 3 | Over 10.0 |
| **Crypt damage (%)** | 0 | None |
|  | 1 | 0.0-10.0 |
|  | 2 | 10.0-20.0 |
|  | 3 | Over 20.0 |
| **Depletion of goblet cells** | 0 | None |
|  | 1 | Mild |
|  | 2 | Moderate |
|  | 3 | Severe |
| **Infiltration of inflammatory cells** | 0 | None |
|  | 1 | Mild |
|  | 2 | Moderate |
|  | 3 | Severe |
